# Triptolide markedly attenuates albuminuria and podocyte injury in an animal model of diabetic nephropathy

**DOI:** 10.3892/etm.2013.1226

**Published:** 2013-07-17

**Authors:** RUIXIA MA, LIQIU LIU, XUEMEI LIU, YAN WANG, WEI JIANG, LUO XU

**Affiliations:** 1 Department of Nephrology, Affiliated Hospital of Qingdao University Medical College, Qingdao, Shandong 266021, P.R. China; 2 Department of Pathophysiology, Qingdao University Medical College, Qingdao, Shandong 266021, P.R. China

**Keywords:** albuminuria, podocyte injury, triptolide

## Abstract

Triptolide is a major active component of *Tripterygium wilfordii* Hook F, which exerts marked immunosuppressive, anti-inflammatory and podocyte-protective effects. In this study, the ability of triptolide to inhibit inflammation and attenuate podocyte injury was examined in a rat model of diabetic nephropathy (DN). Type II diabetic rats with DN were treated with triptolide at a dose of 100 μg.kg^−1^.day^−1^. Following 8 weeks of triptolide treatment, the urine albumin level, kidney weight/body weight and the number of cells positive for ED-1 (a marker for rat mononuclear macrophages) in the kidney were assessed. The effects of triptolide on podocyte injury and chronic inflammation were analyzed using quantitative polymerase chain reaction (qPCR), western blotting and immunohistochemistry. Following triptolide treatment, the albuminuria in the type II diabetic rats was significantly reduced. Furthermore, the glomerular hypertrophy and foot process effacement were improved, and there was a recovery of the slit diaphragm associated with nephrin and podocin expression. The inflammation in the kidneys was also attenuated. Furthermore, triptolide significantly reduced the expression of transforming growth factor-β1 and osteopontin, and the infiltration of ED-1-positive cells into the kidney. The results demonstrated that triptolide markedly attenuated albuminuria and podocyte injury in the rat model of DN, which may have been correlated with the inhibition of inflammation and macrophage infiltration in the kidneys.

## Introduction

Diabetic nephropathy (DN), a major complication of diabetes, is the leading cause of end-stage renal disease worldwide (1). The appearance of microalbuminuria is a detectable early marker of DN. Microalbuminuria may develop into proteinuria and hyperfiltration, followed by reductions in the glomerular filtration rate. The detailed molecular mechanisms underlying the correlation between albuminuria and DN remain elusive. However, functional and structural abnormalities in glomerular podocytes have been recently observed to be one of the earliest events during the development of diabetic glomerular injury (2,3). Podocyte loss and injury are frequently observed in patients with DN at very early stages, which may contribute to the development of severe proteinuria and renal lesions (4–6).

The pathogenesis of DN involves numerous factors, such as metabolic disturbance, abnormal renal hemodynamics and chronic inflammatory factors, including chemokines, adhesion molecules and proinflammatory cytokines (7–9). Chronic inflammation is closely associated with permeability changes in the glomerular filtration barrier and proteinuria in DN (10). Podocytes are located at the outer layer of the filtration barrier, and injury to podocytes is involved in the inflammatory processes of DN (2). It is important to investigate how to prevent podocyte injury in patients with DN; furthermore, novel drugs are required to improve the treatment of podocyte injury in patients with DN.

Extracts of *Tripterygium wilfordii* Hook F (TwHF) have been used in the treatment of glomerulonephritis for >30 years in China. The TwHF extracts have been shown to have significant proteinuria-reducing effects in patients with focal segmental glomerular sclerosis and membranous nephropathy. Triptolide, a diterpene triepoxide, has been identified to be one of the major active components in the TwHF extract. Previous studies have shown that triptolide exerts potent immunosuppressive, anti-inflammatory, anti-proliferative and anti-oxidative effects (11–13). Our previous study and an investigation from another laboratory have indicated that triptolide may inhibit inflammatory responses, thereby reducing albuminuria and improving renal functions in type II diabetic rats and patients with type II diabetes (14,15). However, the possible effects of triptolide on podocytes have yet to be elucidated. In the current study, it was observed that triptolide markedly attenuated albuminuria and improved podocyte injury in a rat model of DN, possibly due to its inhibitory effects on inflammation and macrophage infiltration in the kidneys.

## Materials and methods

### Reagents

Triptolide (molecular formula, C_20_H_24_O_6_) was obtained from the Jiahe Medicine Technology Development Co. Ltd, (Shanghai, China). The purity of triptolide, detected by high-performance liquid chromatography, was 99%. The triptolide was reconstituted in 0.01% dimethyl sulfoxide (DMSO) and freshly diluted with culture medium, prior to use. The final DMSO concentration used in the present study was <0.002% (v/v), which was not harmful to cells. Streptozocin was purchased from Sigma (St. Louis, MO, USA) and then dissolved in citrate buffer (0.01 mol/l, pH 4.5).

### Animals

Fifty 8-week-old male Wistar rats (weight, ~200 g) were purchased from the Laboratory Animal Center of the Qingdao Institute for Drug Control (Qingdao, China). The rats were housed in individual cages in a temperature-controlled room with a 12/12-h light-dark cycle, and were left to acclimatize for 1 week prior to the initiation of dietary intervention. All animal experiments were conducted in accordance with the ethical guidelines of the National Defense Medical College (Qingdao, China) and the EthicsCommittee of Qingdao University Medical College (Qingdao, China) for the care and use of laboratory animals in research.

The rats with type II diabetes were modeled according to the methods previously described by Danda et al (17) and Guo et al (18). Rats were randomly assigned to regular rat chow [n=10, control (NC) group] or a high-fat, high-sucrose diet (n=40; 10% animal fat, 20% cane sugar, 2.5 % cholesterol, 1% cholate and 66.5% regular chow). After 8 weeks, the rats fed on the high-fat, high-sucrose diet were injected intraperitoneally with a low dose of streptozocin (30 mg.kg^−1^). Following this, the type II diabetic rats were divided into two groups, specifically a group without triptolide treatment (n=14, DM group) and a group with triptolide treatment (100 μg.kg^−1^.day^−1^; n=14, DT group). Triptolide was administered intragastrically with a volume <1 ml/day. The drug vehicle DMSO was used as a control. Eight weeks subsequent to treatment, the body weights (BWs) of the rats were examined and urine samples were collected. Following this, the rats were sacrificed and blood samples and kidneys were collected.

### Blood glucose, hemoglobin A1c (HbA1c) and insulin measurements

Blood samples were obtained from the tail veins. Fasting blood glucose (FBG) was determined at 1–2-week intervals in all groups using a glucometer (One Touch™ Surestep™; Lifescan, Inc., Milpitas, CA, USA). The serum insulin level and HbA1c were determined by enzyme-linked immunosorbent assay (ELISA) using antibodies against insulin and HbA1c, respectively (Aquatic Diagnostic Ltd., Glasgow, Scotland).

### Noninvasive blood pressure measurement

Blood pressure was measured using the tail-cuff method and an LE5002 noninvasive blood pressure detecting instrument (Diagnostic Systems Laboratories, Inc., Webster, TX, USA) under resting, conscious conditions in a climate-controlled room (23°C). Five consecutive systolic blood pressure (SBP) measurements were taken.

### Determination of urine albumin and creatinine concentrations

Rats were placed in metabolic cages and their urine was collected for 24 h every 4 weeks for the determination of albumin and creatinine excretion. Urine albumin excretion was determined using a turbidimetric immunoassay kit (Shibayagi Co., Ltd., Shibukawa, Japan). The urine creatinine (Ucr) level was determined using an automatic biochemistry analyzer (model no. 7600-020, Hitachi Ltd., Tokyo, Japan). The urine albumin/Ucr ratio was subsequently calculated.

### Determination of biochemical parameters

The biochemical parameters were determined from the blood samples obtained at the end of 8 and 17 weeks. Serum creatinine (Scr), urea nitrogen, total cholesterol (CH), triglyceride (TG), aspartate transaminase (AST) and alanine transaminase (ALT) levels were determined using the automatic biochemistry analyzer. The creatinine clearance (Ccr) was calculated using the following formula: Ccr = UCr/Scr × V [V, volume of urine per min (ml/min)]. The insulin sensitivity index (ISI) was calculated based on the levels of FBG and INS. The formula was ISI = 22.5/(FBG × INS).

### Transmission electron microscopy

Renal cortex samples, measuring 1 mm^3^, were fixed, embedded and cut into 50-nm sections. The specimens were examined and photographed with a JEM-1200 transmission electron microscope (Jeol Ltd., Tokyo, Japan). The microscopic evaluations conducted are described in the following sections.

### Immunohistochemical staining for nephrin, podocin and ED-1

Renal tissue sections (3 μm) were used for the immunohistochemical staining of nephrin, podocin and ED-1. Deparaffinized sections were stained with primary antibodies, specifically, rabbit-anti-rat nephrin antibody (1:200), podocin antibody (1:200) and goat-anti-rat ED-1 antibody (1:50) at room temperature for 1 h. All antibodies were purchased from Santa Cruz Biotechnology, Inc., Santa Cruz, CA, USA). The color was developed by incubating with diaminobenzidine (Santa Cruz Biotechnology, Inc.) and counterstaining with hematoxylin. Controls were obtained by replacing the primary antibody with phosphate-buffered saline. In total >50 glomeruli and 20 non-overlapping interstitial areas from each section were assessed under high power magnification (x400). The numbers of ED-1 positive cells in each glomerulus and interstitial area were counted and averaged in each group.

### Quantitative polymerase chain reaction (qPCR) analysis

Frozen renal tissues were homogenized and total RNA was extracted with TRIzol reagent (Invitrogen Life Technologies, Carlsbad, CA, USA). The extracted RNA was measured by agarose gel electrophoresis for quality and spectrometry for quantity. A reverse transcription reaction kit [Cat. no. DRR035A; TaKaRa Biotechnology (Dalian) Co. Ltd., Dalian, China] was used.

For fluorescence qPCR, 1,000 ng mRNA sample was added to the reverse transcription system. This was diluted with EASY dilution [TaKaRa Biotechnology (Dalian) Co. Ltd.] in a 10-fold series to generate a standard curve. The qPCR process was carried out in an ABI Prism^®^ 7000 HT sequence detection system (cat. no. 11744-100; Applied Biosystems, Invitrogen Life Technologies). Primers for nephrin, podocin, osteopontin (OPN) and transforming growth factor (TGF)-β1 were designed and synthesized by Shanghai Sangon Biological Engineering Technology Co., Ltd. (Shanghai, China). Each experiment was performed at least three times. The primers used in the PCR process are presented in Table I. A two-step PCR procedure was applied and standard curves for the target genes and an internal reference gene were made under the same conditions. The cycle threshold (Ct) values of the samples were used to calculate the corresponding gene copy number. The results are presented as the ratio of the target gene copy over the housekeeping gene [glyceraldehyde 3-phosphate dehydrogenase (GAPDH)] copy (16).

### Western blot analysis

Cells were lysed in cold cell lysis buffer (50 mM Tris, 150 mM NaCl, 10 mM ethylenediaminetetraacetic acid and 1% Triton X-100) containing protease and phosphatase inhibitors. Briefly, the proteins were separated on 10% sodium dodecyl sulfate-polyacrylamide gel electrophoresis (SDS-PAGE) gels, and subsequently transferred to nitrocellulose membranes. The primary antibodies were nephrin (rabbit anti-rat, 1:4,000), podocin (1:4,000), OPN (goat anti-rat, 1:1,000 and TGF-β1 (mouse anti-rat, 1:1,000) antibodies (Santa Cruz Biotechnology, Inc.). Horseradish peroxidase-conjugated anti-immunoglobulin (Ig) G was used as a secondary antibody (Beijing Zhongshan Golden Bridge Biotechnology Co., Ltd., Beijing, China). The blots were detected using an enhanced chemiluminescence (ECL) system (Beijing Zhongshan Golden Bridge Biotechnology Co., Ltd.).

### Statistical analysis

The data are presented as the mean ± standard deviation of at least three independent experiments. The significance of the differences between the groups was determined by multiple sample comparison methods analysis of variance (ANOVA). P<0.05 was considered to indicate a statistically significant difference.

## Results

### Triptolide significantly decreases urinary albumin levels, kidney weight (KW)/BW and total CH and TG levels

At 8 weeks subsequent to treatment, the numbers of live rats in the NC, DM and DT groups were 10, 11 and 12, respectively (Table II). The renal function and the general parameters of the three groups were evaluated, including urinary albumin, KW/BW, CH levels, TG levels, SBP, FBG and Ccr. There were no statistically significant differences in levels of urea nitrogen, AST and ALT following triptolide treatment. The results from each group are summarized in Table II.

There was no significant change in the BWs of the type II diabetic rats following 8 weeks of triptolide treatment; however, the KWs and the KW/BW in the DM rats were significantly increased compared with those in the NC rats. During the experiment, no significant changes in food and water intake were observed and no diarrhea was noted.

As shown in Table II, the FBG, HbA1c, SBP, insulin sensitivity index, total CH and TG levels in the DM group were higher than those in the NC group (P<0.01–0.05). Moreover, the urinary albumin levels and Ccr in the DM rats were significantly increased compared with those of the rats in the NC group.

Triptolide significantly decreased the urinary albumin levels, KW/BW ratio and total CH and TG levels compared with those in the DM rats (P<0.05). However, no statistically significant differences were observed in SBP, FBG or Ccr between the untreated diabetic and triptolide-treated groups (P>0.05; Table II).

### Triptolide attenuates podocyte injury in rats with DN

**Figure 1:**
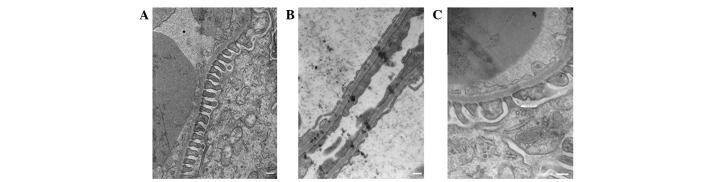
Ultrastructural changes of renal tissue in the (A) control, (B) diabetic and (C) triptolide treatment groups of rats (transmission electron microscopy, bar 200 nm; magnification, ×2,000). The ultrastructural features of the glomerular basement membrane (GBM) thickness, foot process fusion and foot process width were observed under transmission electron microscopy.

To determine the effects of triptolide on renal ultramicrostructure, glomerular podocytes were examined by transmission electron microscopy. As shown in Fig. 1, the foot processes of neighboring podocytes were interdigitated, and the glomerular basement membrane (GBM) thickness was uniform in the NC group (Fig. 1A). However, significant increases in GBM thickness (812.40±65.49 versus 238.64±33.69; P<0.01), foot process fusion rate (56.73±8.40 versus 0; P<0.01) and foot process width (517.87±25.39 versus 261.18±17.55; P<0.01) were observed in the DM rats compared with the NC group (Fig. 1B), which was consistent with the severe albuminuria observed in these rats. Treatment with triptolide was observed to inhibit the thickening of the GBM (276.64±45.42 versus 812.40±65.49; P<0.01), the foot process fusion rate (15.76±6.27 versus 56.73±8.40; P<0.01) and the foot process width (323.14±19.86 versus 517.87±25.39; P<0.01), compared with their values in the DM group, which predominantly normalized the glomerular filtration barrier structure (Fig. 1C).

### Triptolide restores the distribution of nephrin and podocin in rats with DN

**Figure 2:**
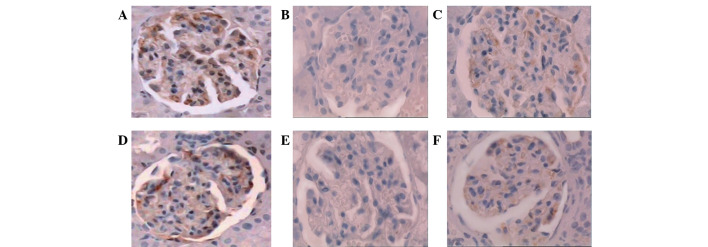
Immunohistochemistry of (A–C) nephrin and (D–F) podocin (hematoxylin staining; magnification, ×400). (A and D) Control group; (B and E) diabetic group and (C and F) triptolide treatment group.

The expression intensity and distribution pattern of nephrin and podocin in glomeruli were observed by immunohistochemical staining. The staining of nephrin and podocin was revealed to form a linear pattern along the glomerular capillary wall in the NC rats (Fig. 2A and D). By contrast, in the DM rats, the expression of nephrin and podocin was decreased significantly (Fig. 2B and E). However, the expression levels of nephrin and podocin in the DT rats were similar to the levels in the NC rats, indicating that triptolide significantly improved the expression of nephrin and podocin in diabetic rats and restored the distribution of nephrin and podocin (Fig. 2C and F).

### Triptolide restores the mRNA expression of nephrin and podocin in renal tissue

**Figure 3:**
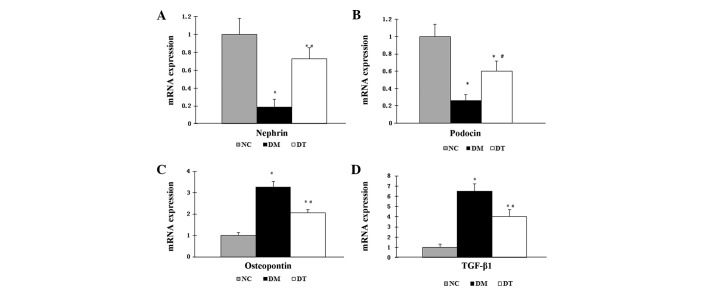
mRNA expression levels of (A) nephrin, (B) podocin, (C) osteopontin (OPN) and (D) transforming growth factor (TGF)-β1 in renal tissue (n=10–12). ^*^P<0.01 compared with the control (NC) group; ^#^P<0.01 compared with the diabetic (DM) group. DT, triptolide treatment group.

The mRNA expression of nephrin and podocin was determined by qPCR. Consistent with the expression intensity of nephrin and podocin, as shown in Fig. 2, the expression levels of nephrin and podocin mRNA were decreased significantly in the kidney cortex of the untreated DM rats compared with the NC rats (Fig. 3A and B, P<0.01). The downregulated expression levels of nephrin and podocin mRNA in the DM rats were restored significantly by the treatment with triptolide (P<0.01; Fig. 3A and B). Thus triptolide treatment restored the mRNA expression of nephrin and podocin in the renal tissue of diabetic rats.

### Triptolide restores the protein expression of nephrin and podocin

**Figure 4:**
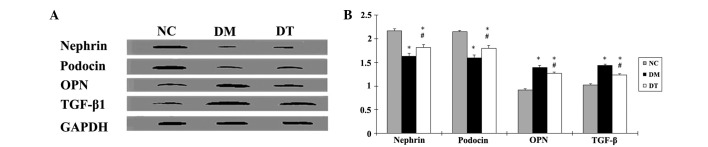
Western blot analysis of the protein levels of nephrin, podocin, osteopontin (OPN) and transforming growth factor (TGF)-β1 in renal tissue (n=10–12). (A) Representative western blotting experiment. (B) Analysis of results from at least three western blotting experiments. ^*^P<0.01 compared with the control (NC) group; ^#^P<0.01 compared with the diabetic (DM) group. DT, triptolide treatment group; GAPDH, glyceraldehyde 3-phosphate dehydrogenase.

Western blot analysis indicated that the expression level of nephrin in the rat kidneys was lower in the DM group than in the NC group (1.63±0.06 versus 2.17±0.02; P<0.05; Fig. 4); similarly, the expression level of podocin in the rat kidneys was also significantly lower in the DM group than in the NC group (Fig. 4B). However, the expression levels of nephrin and podocin in the DT group were significantly higher than those in the DM group (Fig. 4B). Therefore, triptolide restored the expression of nephrin and podocin in the kidneys of rats with DN.

### Triptolide decreases the number of ED-1-positive cells in the glomeruli and interstitium

**Figure 5:**
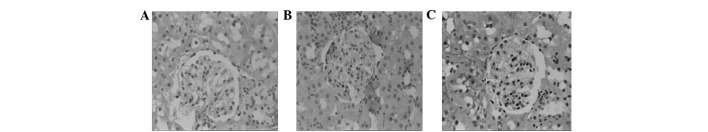
Immunohistochemical analysis of ED-1-positive cells (1:200). (A) Control group; (B) diabetic group and (C) triptolide treatment group. The color was developed by incubating with diaminobenzidine and counterstaining with hematoxylin. Magnification ×200.

Using immunohistochemical staining, the ED-1 expression was examined in each group. The numbers of ED-1-positive cells were significantly increased in the glomeruli of DM rats compared with those of the NC rats (1.96±0.12 versus 0.23±0.06/glomerular cross-section (gcs); P<0.05; Fig. 5). The numbers of the ED-1-positive cells in the interstitium of DM rats were also increased [3.02±0.20 versus 0.41±0.07/high-power field (hpf); P<0.05; Fig. 5]. The increased prevalence of ED-1-positive cells in the glomeruli and interstitium of the DM rats was significantly suppressed by treatment with triptolide (1.32±0.10/gcs in the glomeruli and 2.05±0.19/hpf in the interstitium; Fig. 5). In the DM rats, the correlation between the mRNA expression of nephrin and the number of ED-1-positive cells in the glomeruli was significant (r=−0.696, P<0.05). A similar correlation between interstitial fibrosis and the number of ED-1-positive cells in the interstitium was also observed (r=−0.756, P<0.01). These data suggest that the number of ED-1 positive cells in the glomeruli and interstitium was decreased by triptolide treatment.

### Triptolide suppresses the mRNA expression of TGF-β1 and OPN

The mRNA expression levels of TGF-β1 and OPN in the renal cortex were measured by qPCR. The OPN mRNA expression in the untreated DM rats was increased in comparison with that in the NC rats (P<0.01; Fig. 3C). Treatment with triptolide reduced the OPN mRNA expression by 40% (Fig. 3C). In addition, the TGF-β1 mRNA expression in the renal cortex was significantly increased in the untreated DM rats compared with the NC rats (P<0.01; Fig. 3D). However, this overexpression of TGF-β1 mRNA in the DM rats was significantly suppressed by 56% with triptolide treatment (P<0.01, Fig. 3D). There were significant negative correlations between the mRNA expression of nephrin and OPN (r=−0.794, P<0.01) and between TGF-β1 and nephrin (r=−0.847, P<0.01) in the renal cortex of DM rats. The mRNA levels of TGF-β1 and OPN in the renal cortex of diabetic rats were therefore suppressed by triptolide treatment.

### Triptolide inhibits the protein expression of TGF-β1 and OPN in diabetic rats

Western blot analysis was performed to determine the protein level of TGF-β1 and OPN. The results showed that the protein expression of OPN in the rat kidneys was increased in the DM group, compared with the NC group (Fig. 4B). The overexpression of OPN protein in the diabetic rats was significantly suppressed by treatment with triptolide. The TGF-β1 protein expression in the NC rats was moderate; however, there was an increased expression in the DM rats (1.02±0.03 versus 1.44±0.02; P<0.05; Fig. 4B). The administration of triptolide significantly suppressed the TGF-β1 protein expression. These results suggested that triptolide suppressed the overexpression of OPN and TGF-β1 proteins in the kidneys of diabetic rats.

## Discussion

In the present study, we induced a rat model of DN and studied the effects of triptolide on podocyte injury following 8 weeks of treatment. The results showed that triptolide treatment effectively reduced albuminuria, and ameliorated podocyte foot process effacement and glomerular hypertrophy in diabetic rats. In addition, the recovery from podocyte injury was further demonstrated by increases in nephrin and podocin expression in diabetic rats, following treatment with triptolide. Furthermore, it was observed that triptolide notably decreased macrophage accumulation and the expression of OPN and TGF-β1 in the renal tissue of diabetic rats. The administration of triptolide to the diabetic rats was shown to be safe. During the experiment, the drug did not demonstrate any effect on serum creatinine or urea nitrogen levels in the diabetic rats, and did not exert any adverse effects, such as changes in blood leucocytes, hematocrit or AST or ALT levels, which suggested that triptolide treatment in diabetic patients may be safe, without marrow, liver or kidney toxicity. In combination, the results suggested that the therapeutic effect of triptolide may be achieved by the suppression of macrophage infiltration and OPN and TGF-β1 expression.

It has been suggested that podocyte injury is a major contributor to severe proteinuria in DN. Clinically, the principal features of diabetic podocytopathy manifest as albuminuria and proteinuria (2,3), with the albuminuria and proteinuria resulting from reductions in podocyte number and/or density. These reductions occur due to apoptosis or detachment, GBM thickening with altered matrix composition and a reduction in the expression of nephrin protein in the slit diaphragm, with podocyte foot process effacement. Since podocytes demonstrate a limited ability to proliferate, they are not able to restore the architecture of the GBM, and this condition leads to glomerular sclerosis and tubulointerstitial fibrosis.

Previous studies have revealed that the slit diaphragm protein complex (nephrin-podocin-CD2AP) is an important component in maintaining the glomerular filtration barrier. It has been shown that these podocyte proteins and the onset of proteinuria in type II DN are closely interrelated (19,20). Furthermore, the abnormal expression and distribution of nephrin and podocin have been observed prior to the onset of proteinuria in rat models of DN. A study by Baelde *et al* showed that podocyte loss and significant reductions in the expression of nephrin and podocin in the glomerulus were closely correlated with the occurrence and development of proteinuria (21). In the present study, foot process denudation, GBM exposure and shed podocytes were observed in diabetic rats using transmission electron microscopy. The greater the extent of the podocyte injury, the more the nephrin and podocin expression was downregulated. As a consequence of nephrin and podocin expression downregulation, the structure of the slit diaphragm between the podocytes was destroyed, leading to the onset of proteinuria and subsequent deterioration. All these factors demonstrated that the disorganization of the slit diaphragm between podocytes was capable of accelerating the development of DN. The concept that podocytes are crucial factors in the development of proteinuria and the progression of glomerulosclerosis has indicated that novel approaches are required to treat podocyte lesions. These efforts have led to numerous studies showing that certain reagents, such as angiotensin-converting enzyme inhibitors (ACEIs), angiotensin II receptor blockers (ARBs), sirolimus and darbepoetin, decrease proteinuria by ameliorating podocyte injury (22–24). However, in some patients with severe albuminuria or proteinuria, treatment with ACEI/ARB therapy is not able to produce a satisfactory efficacy. Particular attention has been focused on anti-inflammatory treatments and immunotherapy, which have been shown to attenuate podocyte injury and alleviate albuminuria (25). Chen *et al*(26) demonstrated that triptolide effectively reduced proteinuria and alleviated glomerular immune injuries; furthermore, it markedly improved podocyte lesions and aided the restoration of the normal slit diaphragm structure in rats with passive Heymann nephritis (PHN) (26). In addition to its immunosuppressive and anti-inflammatory activities, the therapeutic effect of triptolide may be due to its direct activity on podocyte injury. Zheng *et al*(27) observed that triptolide protected podocytes from puromycin aminonucleoside (PAN)-induced injury *in vivo* and *in vitro*(27). Furthermore, Gao *et al*(28) revealed that triptolide treatment reduced monocyte chemottractant protein-1 (MCP-1) expression and the number of CD68^+^ macrophages in db/db diabetic mice, with a greater efficacy than valsartan. These studies demonstrated that, as a novel drug, triptolide exerted comprehensive protective effects in the prevention of DN progression (28). In a previous study, we demonstrated that triptolide effectively attenuated proteinuria, suppressed the expression of MCP-1 and OPN and inhibited the infiltration of macrophages in rats with type II DN (15). However, the protective effects of triptolide on podocytes in type II DN remain poorly understood. In the present study, it was demonstrated that triptolide exhibited a favorable anti-albuminuric efficiency and potent therapeutic effect in the recovery from podocyte injury in DN.

In the current study, a type II diabetic rat model was constructed using a high-fat, high-sugar diet, followed by the administration of a low dose of streptozocin (30 mg.kg^−1^) to investigate the mechanism(s) of DN. This rat model successfully imitated human type II diabetes, with moderate hyperglycemia, hypertension, dyslipidemia and insulin resistance. Therefore, it was also an ideal model for studying DN (17,18). In the current study, urinary albumin excretion was significantly higher in the DM and DT groups than in the NC group. In addition, the KW/BW was significantly higher in the DM and DT groups than in the NC group. Podocyte foot process fusion and effacement, in addition to markedly reduced nephrin and podocin expression, were the most predominant characteristics in the DM group, compared with NC group. These results demonstrated that albuminuria and podocyte injuries were closely correlated. In comparison, following 8 weeks of triptolide treatment in the DT group, it was observed that there was a significant reduction in the albuminuria, accompanied by improvements in podocyte foot process effacement and the restoration of nephrin and podocin expression. In combination, these results indicated that the anti-proteinuric effect of triptolide was strongly correlated with recovery from podocyte injury. It has previously been observed that while treatment with an ACEI or an ARB reduced proteinuria, the treatment resulted in significantly decreased SBP and Ccr. By contrast, it was observed in the present study that triptolide treatment had no marked effects on SBP and Ccr levels at the administered dose. Therefore, the results suggested that triptolide reduced proteinuria by the protection of podocytes and the reversal of podocyte injuries in DN rats, rather than by decreasing Ccr, FBP or SBP. This further demonstrates the independent action of triptolide in the reduction of proteinuria. In addition, the correlation between protein intake and proteinuria is a well-known phenomenon in rats. In the present study, triptolide did not affect food intake, which suggested that low protein intake was not responsible for the anti-proteinuric effect.

In this study, we further explored the underlying mechanism by which triptolide exerted the protective effect on podocytes. Previous studies have shown that the progression of DN may be a result of an inflammatory reaction. Furthermore, studies have shown that chemokines, such as interleukin (IL)-1 and TGF-β1, are predominantly synthesized in podocytes and that there are numerous chemokine receptors on the membranes of podocytes. These data suggest that podocytes are important in the inflammatory reaction (29–31). Studies by Takano *et al*(32) and Zhang *et al*(25) showed that active macrophages produced proinflammatory factors, such as IL-1 and tumor necrosis factor (TNF-α), and MCP-1, which reduced the expression of nephrin through the PI3K/protein kinase B (PK-B) and TGF-β signaling pathways, thus influencing podocyte survival (25,32). In the present study, it was shown that triptolide inhibited the production of TGF-β1 and an additional monocyte chemotactic factor, OPN. Moreover, triptolide suppressed the renal infiltration of ED-1-positive cells and decreased urinary albumin excretion in rats with type II DN. These results were consistent with the restoration of podocyte ultramicrostructure and the associated protein expression. In addition, the results indicated that triptolide may alleviate albuminuria and protect podocytes from injury by suppressing the mRNA and protein expression of TGF-β1 and OPN, and inhibiting macrophage infiltration. Although these results suggested that the protective effects of triptolide on podocytes in type II DN may be achieved through an anti-inflammatory mechanism, further studies are required to elucidate the precise signaling pathways, such as the RhoA-and p38 mitogen-activated protein kinase (MAPK)-signaling pathways (33,34). Furthermore, the results of the present study indicate that triptolide is able to attenuate glomerular hypertrophy and improve hyperlipidemia, which may be a contributory factor in the protection of podocytes in DN.

In conclusion, triptolide showed a prominent anti-albuminuric effect in DN. This effect was characterized by an improvement in foot process effacement and the recovery of podocyte injury markers, nephrin and podocin. Notably, triptolide did not ameliorate hyperglycemia, HbA1c levels or SBP in the rats. The therapeutic effect of triptolide may be due to the inhibition of macrophage infiltration, in addition to the decreased secretion of inflammatory cytokines. The results of the study demonstrate that triptolide may provide protective effects in the prevention of DN progression.

## Figures and Tables

**Table I:** Primers used in the study.

Primer	Sequence	Length (bp)
Nephrin (F)	5′-AAGTACGAATGGAC CCCTATGAC-3′	176
Nephrin (R)	5′-CAGGGCTGTAGGAAACGGGTG-3′	
Podocin (F)	5′-CACGGTAGTGAATGTGGACGA-3′	145
Podocin (R)	5′-GAGGA CAAGAAGCCA CTCGCAGGCC-3′	
OPN (F)	5′-TCAGCATTTC GCTTCTGTTCT-3′	111
OPN (R)	5′-CTGTAAGTTTGCCTGCCTCTA-3′	
TGF-β (F)	5′-GCCTGAG TGGCTGTCTTTTGA-3′	197
TGF-β (R)	5′-GGAAGG GTCGGTTCATGTCAT-3′	
GAPDH (F)	5′-TTCTAGAGACAGCC GCATCT-3′	106
GAPDH (R)	5′-TGGTAACCAGG TGTCCGATA-3′	

F, forward; R, reverse; OPN, osteopontin; TGF-β, transforming growth factor-β; GAPDH, glyceraldehyde 3-phosphate dehydrogenase.

**Table II:** General data of the rats used in the study.

	Group		
	
Variable	NC	DM	DT
UAL (mg/mg.Cr)	0.98±0.42	6.36±0.92^a^	2.48±0.57^a^^b^
KW/BW (g/kg)	2.05±0.32	5.06±0.57^a^	2.75±0.61^a^^b^
SBP (mmHg)	111.0±3.8	136.5±5.6^a^	133.8±4.2
FBG (mM)	5.07±1.73	16.41±3.12^a^	18.70±3.56^a^
INS (ng/ml)	18.83±6.20	20.51±7.14	19.26±5.29
ISI	0.182±0.010	0.068±0.015^a^	0.056±0.019^a^
HbA1c	2.89±0.31	6.52±0.48^a^	6.73±0.50^a^
CH (mM)	1.80±0.47	3.87±0.98^a^	2.80±0.56^a^^b^
TG (mM)	0.83±0.31	3.52±0.56^a^	1.62±0.28^a^^b^
Ccr (ml/min)	1.57±0.33	2.28±0.35^a^	1.96±0.57
Urea nitrogen (mM)	8.23±1.25	9.25±2.02	8.28±1.37
AST (U/l)	64.3±12.4	61.6±13.2	60.9±12.6
ALT (U/l)	46.2±7.5	56.2±8.4	58.4±8.7

n=10–12.

a P<0.01 compared with the control (NC) group;

b P<0.01 compared with the diabetic (DM) group.

DT, triptolide treatment group; UAL, urinary albumin; FBG, fasting blood glucose; HbA1c, hemoglobin A1c; SBP, systolic blood pressure; INS, serum insulin; ISI, insulin sensitivity index; CH, cholesterol; TG, triglyeride; KW/BW, ratio of kidney weight to body weight; Ccr, creatinine clearance rate; AST, aspartate transaminase; ALT, alanine transaminase.
